# Supplementary material for: Perceptions of yellow fever emergency mass vaccinations among vulnerable groups in Uganda: A qualitative study
Source: PLoS Negl Trop Dis. 2024 May 13;18(5):e0012173. doi: 10.1371/journal.pntd.0012173 (PMC11115279; doi:10.1371/journal.pntd.0012173)
Supplement: S1 File — (DOCX) [file pntd.0012173.s001.docx]

# **Interview Questionnaire in English**

##### What are the perceptions of YF outbreak from the perspective of experts and affected communities?

- - - What do you think are the causes of YF outbreaks?
    - What are local concepts and the meaning of YF disease and YF outbreak?
    - What is the socioeconomic impact on affected families and communities?

##### What are the perceptions of affected communities towards YF mass immunization?

- - - What are local concepts and experiences of YF mass immunization?
    - What are the implications for national and international interventions?
    - Has there been a prior mass drug application in affected communities?
    - How have prior mass drug applications influenced the current YF mass immunization?

##### What are the perceptions of the affected communities?

- - - What is your experience of the disease?
    - How is the communication between experts and affected communities?

##### What are the differences to the previous YF outbreak and mass immunization in 2010?

- - - Are there differences in local concepts and experiences between outbreak sites in 2010 and 2016?
    - Are there differences in cause of outbreak?
    - Are there differences in local concepts and experiences of YF mass immunization?
    - Does the socioeconomic impact on affected families and communities differ?
    - Does the communication between experts and affected communities at the outbreak sites in 2010 and 2016 differ?

##### What are the environmental related factors for YF outbreak?

- - - Are there environmental related factors (artificial mosquito breeding grounds in urban areas, lack of public sanitation, overcrowded informal settlements…) of YF outbreak?

# **Interview Questionnaire in Luganda**

##### Kiki abakugu nabantu abakosebwa kyebalowoza ku kubalukawo kwobulwade bwo musujja gwe’nkaka?

- - - Kiki kyolowoza ekivako omusujja gwe’kaka okubalukawo?
    - Mulowoza ki ela entegela yamwe kubulwade bwo musujja gwe’keka nokubaluka wo kwabwo eli ki?
    - Omusujja guno gukosa gutya ebyenfuna ne’mbela yabulijo?

##### Kiki abantu abakosebwa obulwade bwo musujja gwe’nkaka kyebalowoza ku kugema kwekikungo?

- - - Mulowoza ki kukugema okwekikungo ela kwabayisa batya?
    - Biki ebitukibwako nga engwanga namawanga amalala genyigide mu kulwanyisa omusujja gwe’nkaka?
    - Wali wabadewo ko okugaba edagala mubintu byona enyakosebwa?
    - Ngeli ki okugaba edagala lye kikungo okwasoka lye kwakosamu okugema kwomusuja gwe’nkaka?

##### Kiki abantu abakosebwa obulwade bwo musujja gwe’nkaka kyebalowoza?

- - - Biki byewayitamu nga olwade obulwade bwo musujja gwe’nkaka?
    - Empulizinganya wakati wabakugu nabantu abalwala omusujja gwe’nka eli etya?

##### Ngyawulo ki eliwo kati woba otunulide ku kubalukawo kwobulwade bwo musujja gwe’nkaka nokugugema mu 2010?

- - - Waliwo engayawulo mundowoza nobumanyi bwabantu wakati wokubalukawo kwo musujja gwe’nkaka mu 2010 ne 2016 mu bitundu ebyakosebwa?
    - Waliwo engyawulo mubiki ebyaleta omusujja gwe’nkaka?
    - Waliwo engyawulo mu ndowoza yabantu nebiki byebayitamu nga bagemedwa omusujja gwe’nkaka?
    - Waliwo engyawulo mungeli abantu gyebakosebwamu mubyenfuna ne mbela yabulijo?
    - Waliwo engyawulo mungeli abakugu nbantu babulijo gye bawuliziganyamu mubitundu ebyakosebwa mu 2010 ne 2016?

##### Bintu ki ebyekusa kubutonde ebyavako okubalukawo kwo musujja gwe’nkaka?

- - - Waliwo ebintu ebyekusa kubyobutonde ne byobuyongyo byemulowoza nti byavako okubalukawo kwo musujja gwe’nkaka

# **Interview Questionnaire in Acholi (Luo)**

##### Tam tye ni ngo ikom two YF ma opoto ni, ki tam ango ma ludiro tye kwede ikom two man i kabedo ma two man opoto iye?

- - - Itamo ni gin ango ma okelo poto pa two YF?
    - Lutedero tamo ni nining ikom tyen two pa YF ki poto ne kany?
    - Peko ango mabino ikom gangi ma two man opoto iye ki kabedo ma dano bedo iye?

##### Tam pa dano ma bedo i kabedo ma two man opoto iye tye ni ngo ikom gwer lumuku pi YF?

- - - Tam pa lutedero ki ngec pi gwer lumuku pi YF tye ni ngo?
    - Peko ma obino calo adwogi pa lobo wa ki lobo ma woko pi lweny i kom two YF tye ni ngo?
    - Tyika tye yat moo ma gimiyo cut cut lumuku i kabedo ma two man opoto iye?
    - Yat magi miyo-oo cut cut lumuku ni, okonyo nining Pi yub me agwera lumuku pi YF?

##### Tam pa dano ma bedo i kabedo ma two YF opoto iye tye ni ngo kono?

- - - Ngec mamegi i kom two man tye ni ngo?
    - Lok ikin ludiro ki dano ma bedo i kabedo ma two YF tye iye, tye nining?

##### Apoka poka ango matye ikom two YF ma opoto macon ki gwer lumuku ma otime i mwaka 2010?

- - - Tyika tye aloka loka me tam ki ngec ikin kabedo ma two man opoto iye i mwaka 2010 ki mwaka 2016?
    - Tyika tye apoka poka ikin jami ma nyayo poto pa two YF?
    - Apoka poka ikom tam pa lutedero ki ngec tye kwene ikom gwer lumuku pi YF?
    - Tyika apoka poka me peko mabino calo adwogi pi two man ikom gangi ma two man opoto iye ki kabedo ma two man bene tye iye?
    - Dong itamo ni gin ango matye i kabedo ma orumu wa matye ka nyayo poto pa two man me YF?

##### Itamo ni tye jami mukene matye i kabedo ma orumi wa matye ka nyayo poto pa two man me YF macalo: bur magi tongo me nywal pa ober matye i dyer taun, onyo, pe pa lengo ma oromo ikin dano?

##### Onyo tore pa dano mapol labongo ngec i kabedo matidi?

# **Interview Questionnaire in Rukiga**

##### Ni nteekateeka ki aha kubarukaho kw’omushwija gw’enkaka kuruga omu nteekateeka y’abakugu hamwe n’abantu b’omubyaro ebinyangaraziibwe omushwija gw’enkaka?

- - - Ni bintu ki ebi orikuteekateeka ngu nibyo birireetaho okubarukaho kw’omushwija gw’enkaka?
    - Ni nteekateeka ki z’abantu b’omubyaro hamwe n’eki endwara y’omushwija gw’enkaka erikumanyisa n’okubarukaho kw’omushwija gw’enkaka?

N’enki ekirikuruga omu mbeera y’entuura y’ekicweka n’entaasya aha maka hamwe n’ebyaro ebinyangaraziibwe omushwija gw’enkaka?

##### Ni nteekateeka ki z’abantu b’omu byaro ebinyangaraziibwe omushwija gw’enkaka aha kugyema omushwija gw’enkaka okwa boona?

- - - Ni nteekateeka ki y’abantu b’ekyaro hamwe n’ebi barabiremu omu kugyema kwaboona kw’omushwija gw’enkaka?
    - Ni bintu ki ebirugire omu mirimo y’eihanga n’ey’aheeru y’eihanga?
    - Hakaba haarabaireho okugaba omubazi okwaboona omu byaro ebinyangaraziibwe omushwija gw’enkaka?
    - Okugaba omubazi okwaboona okw’enyimaho kuhindweire kuta okugyema okwaboona okuriho hati?

##### Ni nteekateekaki y’abantu b’omubyaro ebinyangarazibwe omushwija gw’enkaka?

- - - Iwe endwara egi noogimanyaho ki?
    - Okugabana amakuru ahaagati y’abakugu hamwe n’abantu b’omu byaro ebinyangarazibwe omushwija gw’enkaka kuri kuta?

##### Ni ntaaniso ki eri ahaagati y’okubarukaho kw’omushwija gw’enkaka okuhweire n’okugyema okwa boona okwakozirwe omu 2010?

- - - Hariho entaaniso omu nteekateeka y’abantu b’omubyaro hamwe n’ebi barikumanya aha mushwija gw’enkaka ahaagati y’emyanya ei yaabarukiiremu omu mwaka gwa 2010 hamwe n’omu mwaka gwa 2016?
    - Hariho entaaniso omu birikureetaho okubarukaho kw’omushwija gw’enkaka?
    - Hariho entaaniso omu nteekateeka y’abantu hamwe n’ebi barikumanya aha kugyema kwa boona kw’omushwija gw’enkaka?
    - Embeera erikuruga omu ntuura n’entaasya aha maka hamwe n’ebyaro ebiteganisiibwe omushwija gw’enkaka nibyahukana bita?
    - Okugabana amakuru ahaagati y‘abakugu hamwe n’ebyaro ebinyangaraziibwe omushwija gw’enkaka aha myanya ei gwabarukiiremu omu mwaka gwa 2010 hamwe na 2016 neeyahukana eta?

##### Ni mbeera ki ezaine akakwate n’ebintu ebitwetooroire ahabw’okubarukaho kw’omushija gw’enkaka?

- - - Hariho embeera ezaine akakwate n’ebintu ebitwotooroire (emyaka eteirweho erikukuriramu ensiri omu ndembo, oburofa omubyaro, emyanya erimu abantu baingi …) ezirikureetaho okubarukaho kw’omushwija gw’enkaka?
