# Supplementary material for: Perceptions of yellow fever emergency mass vaccinations among vulnerable groups in Uganda: A qualitative study
Source: PLoS Negl Trop Dis. 2024 May 13;18(5):e0012173. doi: 10.1371/journal.pntd.0012173 (PMC11115279; doi:10.1371/journal.pntd.0012173)
Supplement: S2 File — (DOCX) [file pntd.0012173.s002.docx]

**Yellow fever**

1. What do you know about yellow fever (YF)?
2. Why is it called YF?
3. In your opinion how can you get it?
4. How do you know someone has YF?
5. In your opinion, how can you treat it?
6. How can you prevent getting YF?
7. Are there traditional beliefs about YF?
8. Have you ever had YF?
9. Who can get YF?
10. How is YF different in adults vs. children?
11. How can you distinguish YF from other diseases?
12. Can you have YF without yellow eyes?
    1. Which other diseases can cause yellow eyes?
13. If someone in your family has YF, what would you do?

**Outbreak**

1. What do you know about the YF outbreak?
2. In your opinion what was the cause for the outbreak?
3. How did the outbreak affect your family?
4. How did the outbreak affect daily life?
5. Which precautions did you take?
6. Do you know people, who got sick from YF?
7. In your opinion how did the outbreak affect the community?
8. Please explain how you were informed about YF?
9. In your opinion how was the communication between experts and the community?
10. How did the YF outbreak change your knowledge about YF?
11. If there would be a new outbreak, what would you do?

**Vaccine**

1. What do you know about the yellow fever vaccine?
2. Why did you get vaccinated?
3. How often do you need to get vaccinated?
4. How did you feel about the vaccination?
5. If female and young, were you pregnant when vaccinated? If so, in your opinion how did it affect you and the baby?
   1. How did you feel about getting vaccinated while pregnant?
   2. Any complications during gestation?
   3. Any maternal complications?
   4. Any complications with the baby?
6. Please, could you describe how the vaccination was performed?
7. Why do you receive a certificate?
8. Who in your family got vaccinated?
9. Tell me which impact had the vaccination on your life?
10. In your opinion why would someone not get vaccinated?
11. What are rumors about the vaccine?
12. In your opinion, how did the election period affect the YF mass vaccination?

**Mass vaccination campaign**

1. What is your opinion about mass immunization programs?
2. Please, could you describe your experience?
3. Which prior mass drug application (MDA) have been conducted within your community?
4. If so, in your opinion how has a prior mass drug application influenced the current YF mass vaccination?
5. Would you participate in a future vaccination campaign? Why?

**Environmental related factors**

1. In your opinion what was the cause for the outbreak?
2. In your opinion where can mosquitos breed around here?
3. What do you know about deforestation in your village?
4. Do you have monkeys around?
5. In your opinion how is public sanitation?
6. Do you sleep under a mosquito net?
7. In your opinion what could be improved?
8. In your opinion what could be done in the future to prevent another yellow fever outbreak?
9. Is there anything else you would like to share?

**Demographic**

1. How old are you?
2. Where are you from?
3. Are you married/ traditionally wedded?
4. How many children do you have?
5. What is your level of education?
6. What is your occupation?
7. Do you have any questions?

**Thank you for your time and for sharing your thoughts with us.**
