# Supplementary material for: Perceptions of yellow fever emergency mass vaccinations among vulnerable groups in Uganda: A qualitative study
Source: PLoS Negl Trop Dis. 2024 May 13;18(5):e0012173. doi: 10.1371/journal.pntd.0012173 (PMC11115279; doi:10.1371/journal.pntd.0012173)
Supplement: S3 File — (DOCX) [file pntd.0012173.s003.docx]

**Yellow fever outbreak**

1. Please, tell me about your involvement during the YF outbreak
2. How was the outbreak detected?
3. Which measures were immediately launched?
4. How was the community informed?
   1. Which tools were used?
   2. Could you show me the information material?
5. In your opinion how did the outbreak affect the communities?
6. Which concerns did you have?
7. In your opinion, what are traditional beliefs or YF?

**Mass vaccination campaign**

1. How was the mass vaccination campaign facilitated?
2. How were the communities informed?
3. Who was the target population?
4. How was the vaccine coverage?
5. Where could people get vaccinated?
6. How was the community informed about the vaccine?
7. How did the community respond?
8. How did people respond to the vaccine?
9. Have there been adverse effects of the vaccine?
10. What is your opinion of giving only 1/5^th^ of the dose?
    1. How did the community react to that?
11. Which challenges did you face?
    1. (e.g., political, social challenges, environmental, economic/financial challenges)
12. In your opinion why would someone not get vaccinated?
13. In your opinion, what were rumors about the vaccine?
14. In your opinion, how did the presidential election influence the vaccination campaign?
15. Which measures are planned for the future?
16. After the outbreak, what initiatives have been taken to deal with YF immunization among the newborn?

**Environmental related factors**

1. In your opinion which environmental related factors could have facilitated the YF outbreak?
2. What is your opinion of the unvaccinated refugees from South Sudan in Northern Uganda?
3. In your opinion what could be improved?
4. Is there anything else you would like to share?

**Demographic**

1. What is your designation?
2. How many years of experience do you have?

**Thank you for your time and for sharing your thoughts with us.**
