# Supplementary material for: Perceptions of yellow fever emergency mass vaccinations among vulnerable groups in Uganda: A qualitative study
Source: PLoS Negl Trop Dis. 2024 May 13;18(5):e0012173. doi: 10.1371/journal.pntd.0012173 (PMC11115279; doi:10.1371/journal.pntd.0012173)
Supplement: S4 File — (DOCX) [file pntd.0012173.s004.docx]

**Demographics**

- Age
- Occupation

**Introductory question**

- I am just going to give you a couple of minutes to think about your experience with yellow fever and the emergency mass vaccination during the yellow fever outbreak. Is anyone happy to share his or her experience?

**Questions:**

1. How could you tell that someone has yellow fever?
2. How can one get yellow fever?
3. How can you treat it?
4. Are there any traditional beliefs about yellow fever?
5. What was the cause of the outbreak?
6. Why would you think that the victims in the village were be-witched? (FGD Masaka District)
7. After realizing cases of deaths from yellow fever, why would you as a community accept to get vaccinated since you had realized these deaths occurred from health facilities and hospitals?
8. What are your thoughts on the information and check-ups you received from the medical experts in the district?
9. In case you had other cases of yellow fever what would you do as the community?
10. Which other information would you like to share?

In addition, FGD in pregnant women:

1. Why did you get vaccinated while pregnant when this vaccine is normally not given during pregnancy?
2. Were there any rumors about vaccination during pregnancy?
3. How did the vaccination affect you and your baby?

**Concluding question**

- Of all the things we’ve discussed today, what would you say are the most important issues you would like to express?

**Conclusion**

- Thank you for participating. This has been a very successful discussion
- If there is anything you are unhappy with or wish to complain about, please speak to me
- I would like to remind you that any comments featured in this report will be anonymous

**Thank you for your time and for sharing your thoughts with us.**
