# Supplementary material for: Perceptions of yellow fever emergency mass vaccinations among vulnerable groups in Uganda: A qualitative study
Source: PLoS Negl Trop Dis. 2024 May 13;18(5):e0012173. doi: 10.1371/journal.pntd.0012173 (PMC11115279; doi:10.1371/journal.pntd.0012173)
Supplement: Attachment — Submitted filename: Letter_YF-VAX_Response_to_Reviewers.pdf [file pntd.0012173.s005.pdf]

Dr. med. univ. Lena Huebl
Department of Tropical Medicine,
Bernhard Nocht Institute for Tropical Medicine
& I. Department of Medicine,
University Medical Center Hamburg-Eppendorf,
Bernhard-Nocht-Str. 74
D-20359 Hamburg, Germany
Phone: +49 40 42818-0
Email: l.huebl@uke.de
Web: www.bnitm.de

Hamburg, July 31st, 2023

Dear Editors,

We would like to thank the reviewers for their helpful suggestions that helped to improve the quality of the manuscript PNTD-D-23-00170, entitled "Perceptions of yellow fever emergency mass vaccinations in Uganda: A qualitative study."

We revised the manuscript based on the reviewers' comments. We uploaded a point-by-point response to the reviewers' comments. We uploaded two versions of the revised manuscript, a clean version and a version with changes highlighted in yellow.

We hope that you will consider our revised article for publication in your Journal.

Sincerely,

Lena Hübl

Lena Huebl

Response to Reviewers

Reviewer's Responses to Questions

Key Review Criteria Required for Acceptance?
As you describe the new analyses required for acceptance, please consider the following:

Methods
-Are the objectives of the study clearly articulated with a clear testable hypothesis stated?
-Is the study design appropriate to address the stated objectives?
-Is the population clearly described and appropriate for the hypothesis being tested?
-Is the sample size sufficient to ensure adequate power to address the hypothesis being tested?
-Were correct statistical analysis used to support conclusions?
-Are there concerns about ethical or regulatory requirements being met?

Reviewer #1:
This is a qualitative study exploring a highly critical topic, applying mixed methods for data collection and analyses. The methods section is short and would benefit from more detailed descriptions in several areas:

- It is not clear how participants were selected for either of the data collection methods. The study aims to describe vaccine implementation but has not included any non-vaccinated in the study population. Suggest to describe clearly recruitment, and why, if a purposive sample typical for qual studies, no non-vaccinated were included.
  Thank you for pointing that out. We only interviewed people who were vaccinated. We added a paragraph to clarify this on lines 137-145. We explained participant recruitment in detail on lines 158-166.

- It is not clear how analysis has been done. Inductive content as well as grounded theory are mentioned. The results section is however not including any emerging themes, categories or similar, but is rather a descriptive account of "s/he said…" which depicts it as closer to a presentation of manifest content rather than at analytical level looking at hidden mechanisms. This is specifically evident e.g., for the power perspectives discussed related to political context, which is relying on direct citations from key informants rather than applying a more analytical approach. It seems the various methods and informants included could open up for triangulation and more analytical approaches and my recommendation would be to explore of possible to provide a more elaborated analysis based on that.
  Thank you. We used triangulation to apply a more analytical approach. We added a detailed description of data analysis in lines 223-233. We also added an overview of the coding analysis as seen in Table 1.

- It is also very evident from the lack of non-vaccinated participants, that the interpretations on causes for vaccine refusal or reluctance are based on opinions of the vaccinated participants, and what they think are causes and reasons, this makes the analysis of vaccine barriers somewhat less interesting.
  Thank you for this valid point. We focus less on perceived vaccine barriers. Instead, we elaborated more on the other main categories and added information on power relations between medical experts and the general population.

- I may have missed but couldn't find a section on robustness or reflectivity, neither anything on how and in which language the constant comparisons were done. I note the first and last/main authors are affiliated w European institutions, and a reflective section on how the team has worked together and the outsider/insider roles in analysis would be beneficial.

Thank you for pointing this out. We added a section on robustness and reflectivity, lines 246-275. Constant comparison was done in English. We added this information in lines 227-229.

Reviewer #2:
Overall, the methods section is acceptable in that it is made explicitly clear where the research was conducted, with some additional information helping contextualize the socio-political context of the sites. It's also clear, that appropriate ethical approval was gained.

- However, the specific type of research design adopted should be outlined more clearly at the start of the methods section, especially in terms of how such an approach helped address the overarching research question.
  Thank you, we added this information in lines 126-129.

- Also, more reflection is needed on the relationship between the researchers and the researched – i.e., possible power dynamics in this dynamic need to be reflected upon. Tying in with the latter point, can you state whether any participants left whilst the research was being conducted.
  Thank you for pointing this out. We added a section on reflectivity to elaborate more on power dynamics, as seen in lines 252- 275. We also added information if any participants terminated the interview early lines 200-204.

- It's also worth defining more clearly the specific sampling approach you adopted for this project.
  Thank you, we added information on the field approach and sampling in lines 170-181.

Some more specific points include:

- Line 172 – Can you be more specific relating to who did what during the analysis process (using author initials).
  Thank you, we added this information. Now in lines 227-228.

- Line 174 – Please provide one example from your coding which illustrates this analytical process.
  Thank you, we added Table 1 with an overview of grounded theory coding analysis.

- Line 185 – I don't like the term 'guarantee anonymity' because can this really be the case in any research project? Try rephrasing this using softer terms.
  Thank you, we rephrased it.

- Line 188 – Can you be a little clearer about what exactly participants received (i.e., a gift voucher worth X amount).
  Thank you for pointing this out. We added this information in lines 209-213.

Results
-Does the analysis presented match the analysis plan?
-Are the results clearly and completely presented?
-Are the figures (Tables, Images) of sufficient quality for clarity?

Reviewer #1:
- The results section is per above mostly purely descriptive including citations. No analytical categories are presented. It is a little difficult to assess the results outside the manifest intent of the included citations.
  Thank you for pointing this out. We added analytical categories in lines 278-280. We restructured the entire result section and included more citations for better understanding.

- Results could benefit from sharing the work mentioned done with grounded theory and identification of emerging categories.
  Thank you. We added Table 1 with an overview of grounded theory analysis and Figure 3, a thematic table of the five emerging categories. We hope that this will help to improve the section.

- For the power analyses it would also benefit from understanding better what are direct experiences, norms and thoughts reported, what are those where participants tell "what others think" and what are key informants reflecting from professional perspectives given power of their roles.
  Thank you for pointing this out. We rewrote the entire result section applying this recommendation.

Reviewer #2:
The results section outlines an excellent volume of data that is directly related to the research question. Also, it is good that characteristics of respondents included in the study are clearly outlined at the start of this section.

- However, this section needs more structural work in terms of flowing between themes in helping the reader process what is being said. A thematic table (which includes thematic examples) at the start of this section could help provide such structure.
  Thank you for pointing this out. We added a thematic table (Figure 3). We also restructured the entire result section for a better flow between the themes. We hope this will help the reader to process the topic.

- Also, I was a little bit confused with the theme 'Perception of YF mass immunization', as it thematically mirrors the theme 'Motivation for YF vaccination'. For instance, see primary data cited on line 482 - in terms of meaning can this not relate to motivation against vaccination? Could these themes be integrated somehow?
  Thank you for pointing this out. We restructured the entire result section according to the themes and included this above-mentioned citation in the motivation against vaccination.

Some more specific points include:

- Table 2 (Participants of focus group discussions) – Pregnant women median age cell should be 30.5, not 30,5.
  Thank you, we changed it accordingly.

- Table 3 (Participants of expert interviews) – Relating to occupation, have you spelled out the acronym DHO before using it here?
  Thank you for pointing this out. After revising the manuscript, the acronym DHO is now spelled out in line 172.

- Line 211 – The acronym Village Health Teams has already been spelled out on line 158.
  Thank you, we deleted the spelled-out term in line 211, it is now already spelled out in line 178.

- Line 231 - The acronym Internally displaced people (IDP) has already been spelled out on line 80.
  Thank you, we deleted the spelled-out term in line 231, it is now spelled out in line 81.

- Line 260 – Not sure what you mean by gas here? Do you mean fuel, as in petrol or diesel?
  Thank you for pointing this out. We meant fuel and changed it. It is now spelled out in line 331.

Conclusions
-Are the conclusions supported by the data presented?
-Are the limitations of analysis clearly described?
-Do the authors discuss how these data can be helpful to advance our understanding of the topic under study?
-Is public health relevance addressed?

Reviewer #1:
The conclusions are somewhat supported, but more underscore the experiences of some participants.

- The experiences of others stating vaccine willingness "because they are told to do it" could perhaps be elaborated more.
  Thank you very much for pointing this out. We elaborated on this more and added a section "power relation between health experts and the general population" from lines 620-674.

- Further would find it interesting if conclusions would discuss suggestions beyond community mobilisation, especially related to the political trust- the opposition's reluctance to vaccinate comes of strongly and I would be curious to understand what insightful key informants would suggest related to that.
  Thank you for pointing this out. We added more information on the participatory approach to include local, religious, and political leaders in campaigns, to train HCWs in social skills as they are often deployed to other regions in Uganda, to adapt health education to local needs, to monitor vaccine campaigns, and evaluate them. This information is added in the conclusion and recommendation section, lines 937-972.

Reviewer #2:
The discussion and conclusion sections are appropriate in terms of reflecting on and answering the set research question. Broader implications of findings are also discussed within the context of relevant literature. Further, authors should be particularly commended for their interesting take on the politicization of vaccine campaigns relating to YF immunization, which I feel is very insightful.

- However, a major issue within this section is the lack of reflection on study limitations. More has to be said regarding key methodological considerations. For instance, how transferable are the findings you produced?
  Thank you for pointing this out. We elaborated more on this and added this information in lines 921-934.

- Was any member checking conducted during data analysis and write up?
  Thank you, we did not do any member checking. We added this information in lines 239-240 and in more detail in the limitation section in lines 929-934.

- Is there the possibility that sample characteristics may have shaped your findings in a certain way? More reflection must be provided on these issues.
  Thank you, we added this in the limitation section in lines 921-923. We also elaborated more on it in the reflective section in lines 252-267.

- Also, can the discussion section be split up into different paragraphs to help provide the reader with more structure? Because the section is one 'whole', sometimes it gets a little confusing for the reader. For example, it would make sense to start a new paragraph when you change the topic of discussion, as on line 607.
  Thank you for pointing this out. We added paragraphs accordingly.

Some more specific points include:

- Line 527 – Can you please cite the direct source of this statistic … which I think going by the link you provided here is the world bank.
  Thank you, we added the direct source from the world bank (citation 34), seen in line 797.

- Line 540 – Can you provide a citation or example of how such health education interventions may look like? Do any examples exist from other VHF outbreaks?
  Thank you, we added this information. We explain how health education could look like using an example from Nigeria, lines 810-814. We also elaborate on which positive impact health education had on a community during the Ebola outbreak in Sierra Leone, seen in lines 814-818.

- Line 641 – Can you add a sentence or two here clarifying what exactly you mean … local perceptions rooted to specific community needs … can you support this point with a practical example or citation?
  Thank you, we added this information and explained it with an example from Liberia, seen in lines 958-962.

Editorial and Data Presentation Modifications?
Use this section for editorial suggestions as well as relatively minor modifications of existing data that would enhance clarity. If the only modifications needed are minor and/or editorial, you may wish to recommend "Minor Revision" or "Accept".

Reviewer #1:

- Interview guide/questionnaire should be appended.
  Thank you, for pointing this out. We added them to the supporting material. In S1 the interview questionnaire in English and native languages Luganda, Acholi, and Rukiga. Furthermore, we added the interview guides for the semi-structured interviews (S2), the expert interviews (S3), and the FGDs in S4.

- Use sub-headings in methods to cover critical parts.
  Thank you, we added sub-headings accordingly.

Reviewer #2:

- The overall grammatical/sentence structure of the manuscript is ok, but before publication I think it will have to be much more polished. This point must be stressed to the authors, as it shouldn't take long to go through the manuscript again in making sure the quality of writing is up to the journal's standard.
  Thank you for pointing this out. We polished the manuscript draft.

Summary and General Comments
Use this section to provide overall comments, discuss strengths/weaknesses of the study, novelty, significance, general execution and scholarship. You may also include additional comments for the author, including concerns about dual publication, research ethics, or publication ethics. If requesting major revision, please articulate the new experiments that are needed.

Reviewer #1:
this is a critical area of study and interesting indeed to see results closely linked to mass vacc campaigns. The paper will be able to contribute important knowledge after major revisions per above. The suggested major recommendation could be problematised, ie indeed empowered patients asking questions and seeking information is an ideal situation, and could also be illuminated versus the public health

perspective of disease control, and asking for "unlimited trust" in public measures, eg Covid-19, without vaccination freedom of mobility was challenged etc.

Reviewer #2:
Overall, this is a great contribution to the VHF literature base more broadly, and YF evidence base more specifically. The use of qualitative methods here produced interesting results, and the comparative study design allowed forthe exploration of such results between two different sites. Findings were then interpreted with relevant literature, and implications for practice were sufficiently reflected upon. Although there are areas that need improved (such as overall sentence structure and methodological reflection), this work will be of interest to practitioners and policy makers who specialize in infectious disease, potentially helping inform future vaccination campaigns. For this reason, after the recommended changes have been made, I recommend this manuscript be published.
