# Supplementary material for: Perceptions of yellow fever emergency mass vaccinations among vulnerable groups in Uganda: A qualitative study
Source: PLoS Negl Trop Dis. 2024 May 13;18(5):e0012173. doi: 10.1371/journal.pntd.0012173 (PMC11115279; doi:10.1371/journal.pntd.0012173)
Supplement: Attachment — Submitted filename: Letter_YF-VAX_Response_to_Reviewer_#3.pdf [file pntd.0012173.s006.pdf]

Dr. med. univ. Lena Huebl
Department of Tropical Medicine,
Bernhard Nocht Institute for Tropical Medicine
& I. Department of Medicine,
University Medical Center Hamburg-Eppendorf,
Bernhard-Nocht-Str. 74
D-20359 Hamburg, Germany
Phone: +49 40 42818-0
Email: l.huebl@uke.de
Web: www.bnitm.de

San Antonio, March 31st, 2024

Dear Editors,

We would like to thank the reviewers for their helpful suggestions, which improved the quality of manuscript PNTD-D-23-00170, "Perceptions of yellow fever emergency mass vaccinations among vulnerable groups in Uganda: A qualitative study."

We revised the manuscript based on the reviewer’s comments. We uploaded a point-by-point response to the reviewer’s comments. We uploaded two versions of the revised manuscript: a clean version and a version with changes highlighted in yellow.

We hope you will consider our revised article for publication in your Journal.

Sincerely,

Lena Hübl

Lena Huebl

Response to Reviewer

Key Review Criteria Required for Acceptance?
As you describe the new analyses required for acceptance, please consider the following:

Methods
-Are the objectives of the study clearly articulated with a clear testable hypothesis stated?
-Is the study design appropriate to address the stated objectives?
-Is the population clearly described and appropriate for the hypothesis being tested?
-Is the sample size sufficient to ensure adequate power to address the hypothesis being tested?
-Were correct statistical analysis used to support conclusions?
-Are there concerns about ethical or regulatory requirements being met?

Reviewer #3:

- Lines 137-138: "We only included people who were vaccinated during YF mass vaccination campaigns because the aim was to better understand the perception of YF vaccination" – this doesn't make sense. It would also have been useful to understand the perceptions of those who did NOT accept the vaccination for whatever reasons. This should be recognised as a limitation of this study in the discussion. It is mentioned too briefly in the "limitations" section at the moment. Using this method, it will not be possible to gain a deep understanding of the reasons for vaccine refusal.
  Thank you for pointing this out. We only included vulnerable groups (the elderly and pregnant women), who are typically excluded from YF vaccination except during mass vaccination campaigns. The focus was not on understanding vaccine refusal but on understanding which information reached this vulnerable group and what motivated them to be vaccinated. We added this information in lines 139-142.

  In addition, we added "among vulnerable groups" to the article's title and stated our included population more clearly throughout the manuscript. We hope this will clarify which participants were included in this study.

  The limitation of this study is now also discussed in lines 813-816 and further elaborated on in the limitations section (lines 955-960).

- Lines 139-140: "People who were vaccinated received a YF card. This made it easy to verify who had been vaccinated and at what time." – had everyone kept their YF card carefully? Did nobody lose it?? This sounds improbable to me. So if you are selecting people with a card, you will have a selection bias in favour of individuals who are very well organised and take their health seriously. This should also be recognised as a limitation in the discussion.
  Thank you for pointing this out. Not everyone had a YF vaccination card. The pregnant women in the 2nd study region still had a vaccination card (in this region, mass vaccination had taken place one year before our study). If this was available, it was possible to verify which trimester the vaccination had taken place using the date of the registered vaccination and the current age of the child. Now, in lines 143-148.

- Lines 141-142: "It would have been difficult to detect unvaccinated individuals because our study was conducted several months after the YF mass vaccination, and in northern Uganda even years later." - this does not make sense to me. If you are saying that people can't remember whether or not they had been vaccinated with YF, then how do you think they will be able to answer detailed questions about their perceptions of YF vaccine several years later? I note that the YF vaccination campaign in the North was in 2010 and the study was conducted in 2017. How can

you be confident that the recollections of people will be accurate, 7 years later? I think the authors need to be more self-critical about the reliability of this information.
Thank you for pointing this out. We clarified our study population in lines 148-153. In addition, we now elaborate more on the recall bias in the limitations (lines 952- 955).

- E.g. in the results, line 369-370: "When we were being vaccinated, there wasn't adequate information about yellow fever. They did not give us any information apart from just the call to people to come and get vaccinated". Do you think that this information is reliable, 7 years after the event, in a group of elderly men (whose memory is probably less than perfect)?
  Thank you. The FGD with the elderly men in northern Uganda is just one example. Not knowing what yellow fever is, how it is transmitted, etc., was also widely reported in the semi-structured interviews among elderly men and women, especially in remote regions with limited access to mass media.

- "The focus was not on vaccine refusal, but rather on understanding perceptions of YF vaccination to inform future YF mass immunization and thereby optimize vaccination campaigns." – to me this doesn't make sense. Vaccine refusal is part of the perceptions of vaccination, and is key for informing future campaigns. Surely one of the main aims of future campaigns is to reach the maximum number of people? So how can this be achieved without addressing reasons for vaccine refusal?
  Thank you. As elaborated earlier, we only included vulnerable groups (elderly and pregnant women) who typically only have access to the YF vaccine during mass vaccination campaigns. Now clarified in lines 153- 156.

- Line 161: "were purposively selected from six affected districts" – what was the purposive sampling frame? I can see in table 2 that the median age was 70 which is quite old. Did you have adequate representation of younger age groups too? Table 3 suggests that elderly men were only recruited to an FGD in the north, and pregnant women only in the south. Is it possible that pregnant women in the north may have different views? And elderly men in the south? The languages and cultures are very different.
  Thank you. We interviewed vulnerable groups, the elderly (over 65 years) and pregnant women, who only had access to the YF vaccine during mass vaccination. At study site 1 (northern Uganda, outbreak 2010), we interviewed only elderly men and women. At study site 2 (central and western Uganda, outbreak 2016), we interviewed pregnant women and elderly men and women. We added this information in lines 174-176. Furthermore, we added the overall number of pregnant women in lines 314-315 and Table 2. Although the outbreak in northern Uganda occurred seven years ago, we still sought to include a comparison group within the country. Our study site 1, located in the north, is a vulnerable area with elderly participants and disadvantaged regions that were affected by the civil war.

- Lines 217-218: "The LH transcribed all semi-structured interviews and expert interviews." – Is LH the first author? (in which case, remove "the"). Is she fluent in Acholi, Luganda and Rukiga? Otherwise, how could she transcribe all the interviews?
  Thank you for pointing this out. LH is the first author. She conducted all semi-structured interviews with a field assistant who translated into English. We elaborate on this now in lines 230-234.

- Data analysis: it seems that this was all done by the first author. Were Ugandan colleagues involved in this at all, in any way?
  Thank you, we added this information in lines 240-243 and 245-246.

- Table 1 is very good, but it would be better to place it in the results section rather than the methods. In fact I would suggest it replaces Fig 3 because they both have the same information, and the table is clearer to me than the figure.
  Thank you. In the results section (305-306 and 309), we replaced Figure 3 with Table 1.

- The section on robustness and reflexivity is good. However, it should also consider the extent to which participants were able to recall events 7 years earlier (in the case of Northern Uganda). It should also consider the positionality of Ugandan co-investigators.
  Thank you, we added this information. Now in lines 267-269 and 290-299.

Results
-Does the analysis presented match the analysis plan?
-Are the results clearly and completely presented?
-Are the figures (Tables, Images) of sufficient quality for clarity?

<u>Reviewer #3:</u>

- Lines 345-6: "In addition, posters were displayed at health facilities and vaccination sites in the local language, Acholi, at study site 1 and in English at study site 2."
  - Did you ask participants what they understood from these posters, especially the ones in English? Did they interpret them correctly?

  Thank you. Some participants recalled that posters were visible. The posters in English also included comics. Thus, people could understand them.

- Lines 369 – 381 – you take at face value the quotes and assume it is true that less information was given in the north than in the south. However, an alternative explanation could be that the elderly men in the north had forgotten the details of what happened 7 years ago, whereas the younger women in the south had a much clearer recollection of what had happened the previous year. In fact, the recollection of the elderly men is clearly incorrect, because the elderly women (also in the north) remembered watching an informative film about YF (previous quote)
  Thank you. We also conducted semi-structured interviews with older women from the northern region who had limited access to information. In the FGD with the elderly women from Lamwo, most of them were in IDP camps at the time of the 2010 YF outbreak. These elderly women stated they had YF cases at the camp, and they were actively sensitized by HCWs who visited the IDP camps. The knowledge and access to information varied greatly, even within the districts.

- Also – good information was not universal in the south – as demonstrated by the quote in lines 775-779
  Thank you for pointing this out. This interview was conducted with a young woman from a remote village in Kalangala (Ssese Islands) who had no access to mass media. In both study sites particularly people from remote villages lacked access to information.

- Line 499 and following– from which study site is the female health worker with 2 years' experience? Would be good to state this for all the medical experts.
  Thank you. The female HCW is from Rukungiri. We added the districts to all quotes.

- Lines 581-583 – this quote is strange. It is from an 84-year-old man referring to his children being at boarding school. Does he mean his grandchildren or even great-grandchildren? His children must be adults!
  Thank you, we clarified it. It's his great-grandchildren. Now in line 593.

- Line 738 – there is a mistake here. It should be NRM not NRF, and it stands for National Resistance Movement - https://www.nrm.ug/

Thank you. You are right; we have changed it accordingly. Now, in line 762.

- Line 740 – should this be RDC not FDC? If it is FDC, what does this stand for?
  Thank you. FDC stands for "Forum for Democratic Change" (lines 758-759) and is the main opposition party in Uganda. https://fdc.ug/

Conclusions
-Are the conclusions supported by the data presented?
-Are the limitations of analysis clearly described?
-Do the authors discuss how these data can be helpful to advance our understanding of the topic under study?
-Is public health relevance addressed?

Reviewer #3:
- Lines 949 – 951: "Vaccination campaigns can therefore never be considered independent of political systems and power relations. Thus, we recommend that awareness campaigns focus on understanding the importance of vaccination rather than on having a YF certificate for travel".

  - The first sentence seems to be an over-generalisation – this seems a very bold statement, and I'm not sure it can be justified on the basis of this small study in a very specific context. For example, I doubt that childhood vaccinations are seen as part of the political system in most countries. In the example of COVID vaccines, while there were all sorts of conspiracy theories, not everyone believed in them.

  - Why not include awareness in the campaigns of the real reason why the card is yellow, and that this is an international standard (not only in Uganda)?

  Thank you. We changed it accordingly. Now, in lines 989-992.

- Lines 957 – 8: "For example, in Liberia after the Ebola outbreak, when routine immunization was reintroduced." This is not a complete sentence.
  Thank you, we completed the sentence. Now, in lines 998-1001.

- Lines 962 – 4: "We also recommend that two conflicting campaigns, whether another immunization campaign or an election campaign, should not be conducted at the same time or in close proximity." – that's all very well in theory, but what do you do if you get a YF outbreak just after you've finished another vaccination campaign, and it happens to also be an election year? One cannot plan for outbreaks in advance, and surely it is still better to conduct the mass immunisation than not to do it.

  - So – rather than saying not to do this, perhaps this should just be a recommendation for better information to explain the reasons.

  Thank you, we changed it accordingly. Now in lines 1003-1005.

Editorial and Data Presentation Modifications?
Use this section for editorial suggestions as well as relatively minor modifications of existing data that would enhance clarity. If the only modifications needed are minor and/or editorial, you may wish to recommend "Minor Revision" or "Accept".

Reviewer #3: (No Response)

Summary and General Comments

Use this section to provide overall comments, discuss strengths/weaknesses of the study, novelty, significance, general execution, and scholarship. You may also include additional comments for the author, including concerns about dual publication, research ethics, or publication ethics. If requesting major revision, please articulate the new experiments that are needed.

Reviewer #3:

I note that this version already incorporates revisions in response to previous reviews. I have not reviewed this manuscript before, so I apologise if some of these suggestions were not made previously. Overall this article is well-written and deserves to be published.

The major issues are

1. Sampling bias – only vaccinated people with a card were interviewed
2. Recall bias – participants in the North were interviewed 7 years after the vaccination! And they were mainly elderly people with less than perfect memories. This should be recognised as a major limitation.
3. Reflexivity - this section focuses only on the first author. The positionality of Ugandan co-investigators and colleagues should also be mentioned.

It is now too late to address the bias in the design, but it should be discussed more extensively in the discussion, and the authors should recognise that this may influence their interpretation of any differences between the North and South – which may be partly due to greater recall bias in the North.

Thank you for pointing out these three major issues. We addressed them accordingly in the revised version. Limitations (952-960), Discussion (815-820), Reflexivity (267-269 and 290-299), and study design (139-142).

Specific comments

Abstract:

- Methodology – add a sentence about the analytical method
  Thank you, we added this information in lines 29-31.

- Conclusion – should be "We therefore recommend…" – (remove commas)
  Thank you, we changed it accordingly. Line 41-42.

Intro:

- Line 94-95: "the YF vaccine is planned to be introduced into the routine immunization program in mid- 2022" – please update to state whether or not this has happened. 2022 is now in the past not in the future!!
  Thank you, we updated this information. YF vaccine has been implemented into routine immunization in October 2022, followed by phased mass vaccination campaigns in 2023 and 2024. Now, in lines 94-99.

Discussion:

- Line 881 – you suggest a film but in fact quotes above say that this was already done. Perhaps the discussion should consider how such films could be shown more widely to reach more people.
  Thank you. We elaborated on this now in lines 841-844.

- Line 813 – you mention a booklet to take home – but is this likely to help in areas with such high levels of illiteracy as you have just stated above?
  Thank you. The booklet could be comics. We added this information in lines 844-846.

- Lines 821-822 - “Participants complied with this request without question. This may indicate that HCWs are trusted as experts, but it also reflects a power imbalance that may backfire in future campaigns”- this is because you only interviewed people who had been vaccinated! So, what it really reflects is sampling bias in your sample!
  Thank you for pointing this out. As elaborated earlier, our study only included vulnerable groups (elderly over 65 and pregnant women) who were vaccinated. The aim was to explore vaccine perception among these vulnerable groups who usually don’t have access to the YF vaccine except during mass vaccination.

- Lines 856 – 858: “Furthermore, in Zambia, community members and HCWs perceived that Western countries brought COVID-19 vaccines to eliminate the African population and that certain vaccines were given to Africans who were rejected by Western countries.” – is it the Africans who were rejected by Western countries, or the vaccines?
  Thank you. We meant the vaccines were rejected. We clarified it now in line 890.

- Lines 911-912: “This had disastrous consequences with unwanted teenage pregnancies due to fertility testing and increased infertility due to botched abortions” – this doesn’t make sense. Why would fertility testing cause unwanted teenage pregnancies?
  Thank you, we clarified it now in lines 943-947.

- Line 924: “social incompetence in the public sector” – what do you mean by this?
  Thank you, we added an explanation. Now in lines 963-965.
